# Cuproptosis-related LncRNAs are potential prognostic and immune response markers for patients with HNSCC via the integration of bioinformatics analysis and experimental validation

**DOI:** 10.3389/fonc.2022.1030802

**Published:** 2022-12-22

**Authors:** Liuqing Zhou, Qing Cheng, Yao Hu, Haoyue Tan, Xiaoguang Li, Shuhui Wu, Tao Zhou, Jieyu Zhou

**Affiliations:** ^1^ Department of Otorhinolaryngology, Union Hospital, Tongji Medical College, Huazhong University of Science and Technology, Wuhan, China; ^2^ Department of Otorhinolaryngology, The Central Hospital of Wuhan, Huazhong University of Science and Technology, Wuhan, China; ^3^ Department of Otorhinolaryngology-Head and Neck Surgery, Shanghai Ninth People’s Hospital, Ear Institute, Shanghai Key Laboratory of Translational Medicine on Ear and Nose Diseases, Shanghai Jiaotong University School of Medicine, Shanghai, China; ^4^ Department of Otorhinolaryngology, Baoshan Branch, Shuguang Hospital Affiliated with Shanghai University of Traditional Chinese Medicine, Shanghai, China

**Keywords:** cuproptosis, long noncoding RNA, HNSCC, TCGA, immune infiltration, prognosis

## Abstract

**Introduction:**

Head and neck squamous cell carcinoma (HNSCC) is a malignant neoplasm typically induced by alcohol and tobacco consumption, ranked the sixth most prevalent cancer globally. This study aimed to establish a cuproptosis-related lncRNA predictive model to assess the clinical significance in HNSCC patients.

**Methods:**

The Cancer Genome Atlas (TCGA) database was utilized to download cuproptosis-related genes, lncRNAs profiles, and selected clinical information of 482 HNSCC samples. Cuproptosis-related lncRNAs were analyzed by Pearson correlation method, with the least absolute shrinkage and selection operator (LASSO) and univariate/multivariate Cox analyses performed to establish the cuproptosis-related lncRNA predictive model. Subsequently, the time-dependent receiver operating characteristics (ROC) and Kaplan-Meier analysis were applied to assess its prediction ability, and the model was verified by a nomogram, univariate/multivariate Cox analysis, and calibration curves. Furthermore, the principal component analysis (PCA), immune analysis, and gene set enrichment analyses (GSEA) were performed, and the 50% inhibitory concentration (IC50) prediction in the risk groups was calculated. Furthermore, the expression of six cuproptosis-related lncRNAs in HNSCC and paracancerous tissues was detected by quantitative real-time PCR (qRT-PCR).

**Results:**

A total of 467 lncRNAs were screened as cuproptosis-associated lncRNAs in HNSCC tissues to establish an eight cuproptosis-related lncRNA prognostic signature consisting of AC024075.3, AC090587.2, AC116914.2, AL450384.2, CDKN2A-DT, FAM27E3, JPX, and LNC01089. For the high-risk group, the results demonstrated a satisfactory predicting performance with considerably worse overall survival (OS). Multivariate Cox regression confirmed that the risk score was a reliable predictive factor (95% CI: 1.089–1.208, hazard ratio =1.147), with the area of 1-, 3-, and 5-year OS under the ROC curve of 0.690, 0.78524, and 0.665, respectively. The differential analysis revealed that JPX was significantly upregulated in HNSCC tissues, while AC024075.3, AC090587.2, AC116914.2, AL450384.2, CDKN2A-DT were downregulated in HNSCC tissues by qRT-PCR assays. In addition, this gene signature was also associated with some immune-related pathways and immune cell infiltration and affected the anti-cancer immune response. Furthermore, Bexarotene, Bleomycin, Gemcitabine, etc., were identified as potential therapeutic compounds for HNSCC.

**Discussions:**

This novel cuproptosis-related lncRNAs prognostic signature could predict prognosis and help propose novel individual therapeutic targets for HNSCC.

## Introduction

Head and neck squamous cell carcinoma (HNSCC) is among the main cancer-related mortality causes globally, with an estimated 700,000 cases annually (1). Despite improved detection and therapy techniques, HNSCC patients’ 5-year overall survival remains 40-50%, with substantially lower rates in advanced cancer patients (2). The main reasons for high fatality rates are late detection, fast development, and poor treatment outcome (3). Although the molecular alterations that drive neoplastic transformation and tumor progression in HNSCC has been growing rapidly, very few biomarkers are currently used in clinical practice or have proceeded towards validation for routine use. Accordingly, more efficient biomarkers for early detection of HNSCC is the clinical required.

Trace elements, including copper (Cu), iron (Fe), and zinc (Zn), are important components of biological systems and regulate functions in many biochemical processes including mitochondrial respiration, iron uptake, antioxidant/detoxification processes, and biological pathways regulation. It has been shown that disturbances in Cu homeostasis may lead to structural abnormalities or loss of some essential physiological functions (4, 5). There is evidence that Cu homeostasis is deregulated in many cancers, so targeting Cu has been considered a new approach for treating cancer (6, 7). Treatment with inhibitors of other known cell death mechanisms including ferroptosis, necroptosis, oxidative stress all failed to abrogate copper ionophore-induced cell death.

Cuproptosis, proposed by Tsvetkov and colleagues, is a novel process of programmed cell death distinguished from oxidative stress-related cell death (e.g., ferroptosis, apoptosis, and necroptosis). The copper-induced cell death is mediated by an ancient mechanism: protein lipoylation (8, 9). Recent studies have demonstrated that triggering cuproptosis has an anti-cancer potential (10). Proteins such as ATP7A (11), ATP7B (12), CDKN2A (13), and NLRP3 (14) can accelerate tumor cell proliferation and metastasis, therefore are defined as cuproptosis-associated proteins.

Long noncoding RNAs (lncRNAs) have no ability for translating into proteins but are involved in transcriptional and post−transcriptional regulation and may serve as oncogenes or tumor suppressors in many cancers, such as HNSCC (15–17). In addition, lncRNAs also play an important role in Cu metabolism as epigenetic regulators, so they could be used to help identify tumor progression (18). Hence, it is important to acquire more cuproptosis-related lncRNAs knowledge for HNSCC diagnosis, prognosis, and treatment.

This study utilized the TCGA to acquire the RNA-sequencing and clinical data to construct a cuproptosis-related lncRNAs model, providing a valuable source of information for the prognosis, sub-type determination, immunity regulation, and possible therapeutic efficacy for HNSCC patients.

## Methods

### Data collection

The TCGA was utilized to obtain the RNA-sequencing, clinical, and mutation data, as well as demographic and clinical data such as sex, age, survival status, TNM classification, stage, and survival outcomes (https://portal.http://gdc.cancer.gov).

### Selection of cuproptosis-related genes and lncRNAs

The cuproptosis-related genes and lncRNAs were obtained from the TCGA database. Nineteen cuproptosis-related genes, including NFE2L2, NLRP3, ATP7B, ATP7A, SLC31A1, LIAS, FDX1, LIPT1, LIPT2, DLD, PDHA1, DLAT, MTF1, PDHB, GLS, DBT, CDKN2A, GCSH, and DLST were retrieved from TCGA according to previous studies (9, 19, 20). The cuproptosis-related lncRNAs were detected *via* the Pearson’s correlation analysis following this criterion, *p*<0.001 and |Pearson R| >0.3.

### Risk model construction and verification

The samples were randomly classified into two risk groups (1:1, training and testing) by the Strawberry Perl and caret R package (R version 4.0.5). The purpose of the training set was to build a cuproptosis-related lncRNAs model, whereas the testing set was used to evaluate the established model performance. No significant differences were identified in the clinical data between the sets.

TCGA dataset was carefully screened for cuproptosis-related lncRNAs prognosis and HNSCC survival data by univariate Cox regression analysis from 467 cuproptosis-related lncRNAs (*p*<0.05). The LASSO Cox regression application (using the 10-fold cross-validation for estimating the penalty parameter) by the R package glmnet identified 15 cuproptosis-related lncRNAs linked to HNSCC OS, which were analyzed by multivariate Cox regression.

The risk model was established using eight cuproptosis-related lncRNAs, and the risk score was computed as follows: $risk\;score=\sum_{k=1}^{n}coef(lncRNA^{k})*expr(lncRNA^{k})$ , coef (lncRNA) is the lncRNA coefficient correlated with survival, and expr (lncRNA) means lncRNA expression. The median risk level was considered a cut-off point, and two risk categories were established, lower and higher risk (21).

### Functional analysis

The differentially expressed genes were detected using the Gene Ontology (GO) analysis *via* the clusterProfiler R package and exhibited statistical significance when p-value< 0.05.

### The immunotherapeutic treatment models

The tumor mutational burden (TMB), the total tumor-specific amount of mutated genes, is linked to neoantigen formation, which triggers immunity against tumors (22). The evaluation and the mutation data sum were conducted by R package maftools. The immunotherapeutic prediction was achieved using Tumor Immune Dysfunction and Exclusion (TIDE) prediction score (23), then TIDE algorithm was applied to assess similarity of immunotherapeutic response in HNSCC.

### Kaplan-Meier survival analysis and principal component analysis (PCA)

PCA was employed to decrease the dataset dimensionality, define hierarchical groupings, and depict the high-dimensional distribution of all samples across all gene expression profiles, risk model, cuproptosis genes, and cuproptosis-related lncRNAs. The survMiner and survival R packages, with the aid of Kaplan-Meier survival analysis, were utilized for determining the OS differences among the two risk groups.

### Nomogram and calibration

The rms R package was used to develop a nomogram for the 1-, 3-, and 5-year OS forecast depending on tumor grade, gender, age, tumor stage, and risk score. The actual and anticipated results consistency was evaluated by calibration curves according to the Hosmer-Lemeshow test.

### Gene set enrichment analyses (GSEA)

The enriched pathways in the two risk groups were identified by the GSEA software using the curated gene set (kegg.v7.4.symbols.gmt), following the criteria: FDR< 0.25 and *p*< 0.05.

### The tumor immune microenvironment (TIME) and immune checkpoints

The software TIMER, XCELL, CIBERSORT, EPIC, MCPcounter, QUANTISEQ, and CIBERSORT on TIMER 2.0 were utilized for the immune cell factors analysis of the two risk groups per the GSEA results. The HNSCC patients’ immune infiltration status was calculated, and the TCGA tumor infiltration estimation profiles were downloaded from the similar website. Wilcoxon signed-rank test, scales, limma, ggtext, and ggplot2 R packages were used to analyze differences in immune infiltrating cells, as presented in the bubble chart. A comparison of the risk groups’ immune checkpoint activation and TIME scores was conducted *via* the ggpubr R package.

### The chemosensitivity prediction model

The R program pRRophetic was applied to examine each HNSCC patient’s chemosensitivity treatment response using the half-maximal inhibitory concentration (IC50) in the Genomics of Drug Sensitivity in Cancer database (GDSC).

### Expression of cuproptosis-associated lncRNAs in tissues by qRT - PCR

A total of 16 matched HNSCC and paracancerous tissues were obtained from Wuhan Union Hospital. Pathologists histopathologically confirmed the diagnosis of all tissues. All patients had not received chemotherapy, radiotherapy, targeted drugs, immunotherapy, or Chinese herbal medicine. Patients were not diagnosed with malignancy at other sites or with other serious underlying diseases. The Ethics Committee of Wuhan Union Hospital authorized this research. All patients signed the informed consent form before surgery. The specimens were removed and rapidly frozen in liquid nitrogen and stored in a low-temperature refrigerator at -80°C for subsequent studies. Total RNA was extracted from samples with TRIzol™ Reagent (Invitrogen). RNA was reverse-transcribed using PrimeScript™ RT Master Mix (Takara). Real-time PCR was performed with TB Green™ Premix EX Taq™ II (Takara). The expression levels of lncRNAs were normalized by GAPDH. The relative expression was calculated based on the comparative Ct (2−ΔΔCt) method, and Student’s t-test (two-tailed) was utilized to assess the significance of lncRNA expression differences in GraphPad Prism (version 8.0). The sequences of primers were as follows:

AC024075.3
Forward: 5′ -CCTGTCAGTTATGCGAAGATGC -3′
Reverse: 5′ -AGGGCATTCCAAGCACAAAAG -3′
AC090587.2
Forward: 5′ -TCATTCTCAAGTCGCTGACACCT -3′
Reverse: 5′ -TGGTGGGAAAATCAGCAAACT -3′
AC116914.2
Forward: 5′ -GACCTCACAAATGTCACCTGGAT -3′
Reverse: 5′ -CACGATCTCTACTCACTGCAACCT -3′
AL450384.2
Forward: 5′ -TCAGTCCTCTGATCACCTTGAGAAC -3′
Reverse: 5′ -TCCACTGCTTTACGACCCATT -3′
CDKN2A-DT
Forward: 5′ -ACAATCCCACTGTGGCAGAGAA -3′
Reverse: 5′ -CCAGGTAACTGAATCCAGCCAA -3′
JPX
Forward: 5′ -ACAAAGTATGAGAACTGAGCCTGAA -3′
Reverse: 5′ -TTCCTCTTCTCGGTGCCTAATC -3′

### Statistical analysis

Bioconductor packages in R, version 4.0.2 software, were employed for statistical data analysis. ROC curve was deployed for evaluating predictive ability of HNSCC prognostic and other clinicopathological signatures *via* the “timeROC” R package. Univariate and multivariate Cox proportional hazard regression analyses were performed to confirm OS clinical characteristics independent prognostic value. Kaplan-Meier method was applied to assess the cuproptosis-related lncRNA signature survival prediction in HNSCC patients. A *p-value*<0.05 indicated statistical significance.

## Results

### Identification of differentially expressed cuproptosis-related lncRNAs and risk model construction for HNSCC

**Figure 1:**
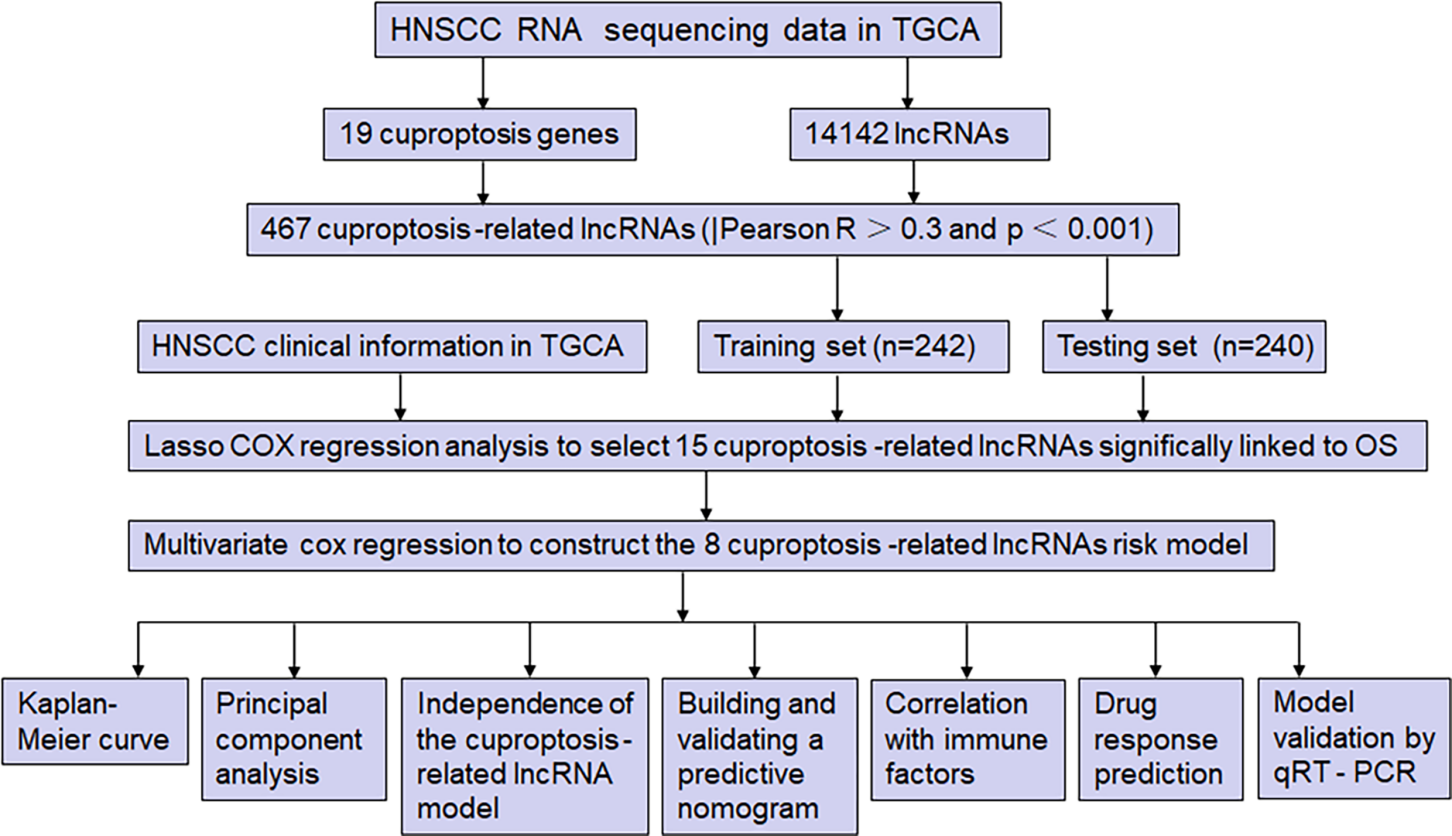
The study flow diagram. The TCGA database was used to obtain 19 cuproptosis-related genes matrix expression data and 14,142 lncRNAs data, and visualization of cuproptosis-related lncRNAs coexpression network is presented in Sankey diagram. In total, 467 lncRNAs underwent screening as cuproptosis-associated lncRNAs, and univariate Cox regression analysis disclosed that 32 cuproptosis-related lncRNAs in the training set and OS were significantly correlated. Then, LASSO regression was employed to detect 15 cuproptosis-related lncRNAs for multivariate analysis. Finally, eight cuproptosis-related prognostic lncRNAs (AC024075.3, AC090587.2, AC116914.2, AL450384.2, CDKN2A-DT, FAM27E3, JPX, and LNC01089) were identified as cuproptosis-related lncRNAs. Cuproptosis-related lncRNAs were analyzed by Pearson correlation method, with the LASSO and univariate/multivariate Cox analyses performed to establish the cuproptosis-related lncRNA predictive model. Subsequently, the time-dependent ROC and Kaplan-Meier analysis were applied to assess its prediction ability, and the model was verified by a nomogram, univariate/multivariate Cox analysis, and calibration curves. Furthermore, the PCA, immune analysis, and GSEA were performed, and the IC50 prediction in the risk groups was calculated. Furthermore, the expression of six cuproptosis-related lncRNAs in HNSCC and paracancerous tissues was detected by qRT-PCR.

**Figure 2:**
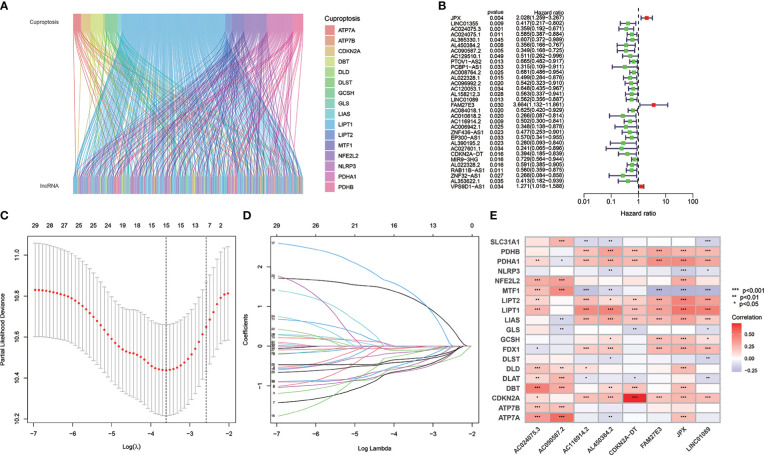
The cuproptosis-related lncRNAs identification and risk model for HNSCC patients. **(A)** Nineteen cuproptosis genes and cuproptosis-related lncRNAs are shown in the Sankey relational diagram. **(B)** Univariate Cox regression was conducted for assuring the selected lncRNAs and figuring out the correlation with clinical prognosis. **(C, D)** LASSO regression analysis following minimum criteria. **(E)** A heatmap intended to express the relationship between 19 cuproptosis genes and the eight cuproptosis-related lncRNAs.

**Figure 1** shows the risk model construction process and the collective analyses. The TCGA database was used to obtain 19 cuproptosis-related genes matrix expression data and 14,142 lncRNAs data, and visualization of cuproptosis-related lncRNAs coexpression network is presented in Sankey diagram (**Figure 2A**). In total, 467 lncRNAs underwent screening as cuproptosis-associated lncRNAs, and univariate Cox regression analysis disclosed that 32 cuproptosis-related lncRNAs in the training set and OS were significantly correlated (**Figure 2B**). Then, LASSO regression was employed to detect 15 cuproptosis-related lncRNAs for multivariate analysis (**Figures 2C, D**). Finally, eight cuproptosis-related prognostic lncRNAs (AC024075.3, AC090587.2, AC116914.2, AL450384.2, CDKN2A-DT, FAM27E3, JPX, and LNC01089) were identified as cuproptosis-related lncRNAs to explore the correlation between cuproptosis-related genes and cuproptosis-related lncRNAs (**Figure 2E**).

### The risk model construction and validation

**Figure 3:**
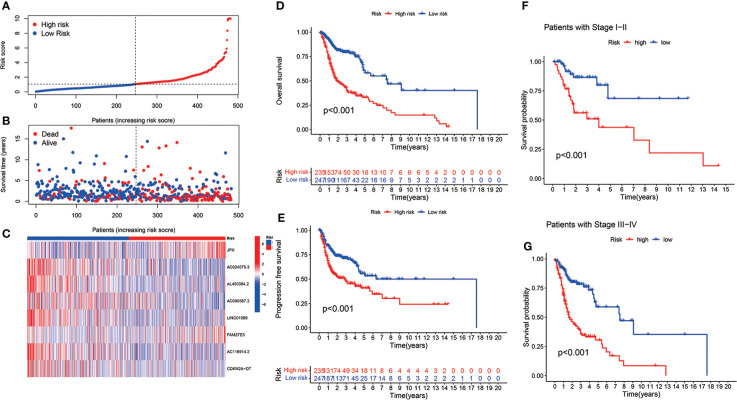
The eight cuproptosis-related lncRNAs prognostic value in TCGA entire cohorts. The survival time and risk score correlation analysis **(A)** The cuproptosis-related lncRNA model-based risk score distribution. **(B)** The survival status and time differences among both groups. **(C)** The eight prognostic lncRNAs expression standards for every patient are shown in the clustering analysis heatmap. **(D, E)** OS and PFS Kaplan-Meier survival curves for both groups of patients. **(F, G)** In TCGA entire cohorts, OS differences in Kaplan-Meier curves stratified by stage between the two risk groups.

**Figure 4:**
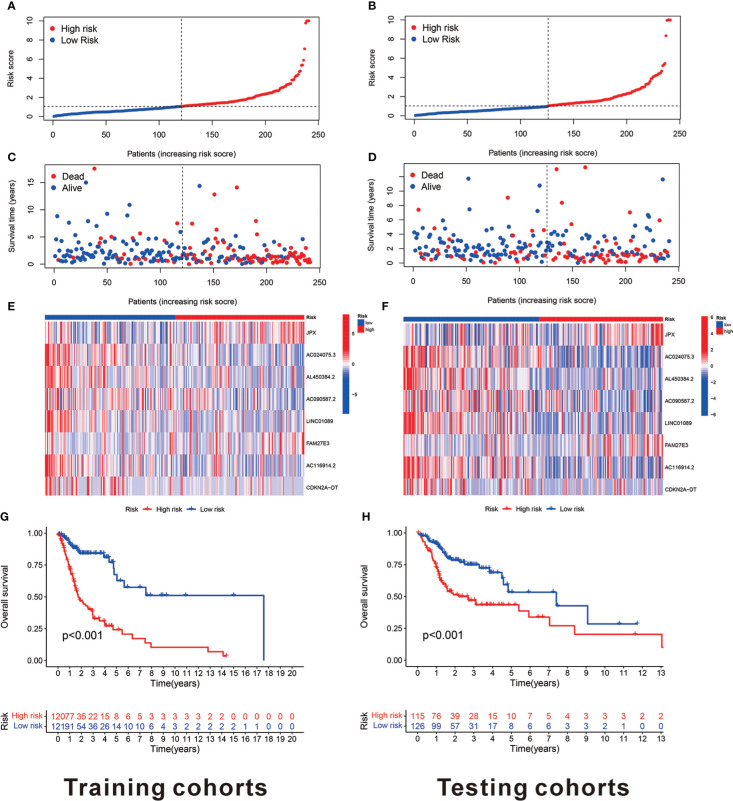
The eight cuproptosis-related lncRNAs prognostic value in TCGA training and testing cohorts. **(A)** The cuproptosis-related lncRNA model-based risk score distribution for training cohorts. **(B)** In training cohorts, the two risk groups’ survival time and status patterns. **(C)** Clustering analysis heatmap reveals that eight prognostic lncRNAs display levels for every patient in the training cohorts. **(D)** In the training cohorts, the two groups OS Kaplan-Meier survival curves. **(E)** The cuproptosis-related lncRNA model-based risk score distribution for testing cohorts. **(F)** In testing cohorts, the two risk groups’ survival time and status patterns. **(G)** Clustering analysis heatmap reveals 12 prognostic lncRNAs expression levels for every patient in testing cohorts. **(H)** In the testing cohorts, the two groups OS Kaplan-Meier survival curves.

The risk model was established using eight cuproptosis-related lncRNAs to estimate the HNSCC individuals’ prognostic risk. All of them were independently associated with OS. On the basis of median risk value, HNSCC samples were classified into higher- and lower-risk groups. The risk grades distribution, cuproptosis-related lncRNAs expression, and survival status and time patterns in the entire set are shown in **Figures 3A–C**. Kaplan-Meier survival analyses were conducted to assess HNSCC patients’ progression-free survival (PFS) and OS, revealing that the high-risk group had worse scores (**Figures 3D, E**). Furthermore, a subgroup analysis was conducted by the log-rank test of people suffering from various stages of HNSCC, showing that for the patients in stages I–II and III–IV, the low-risk group patients had better OS (**Figures 3F, G**). **Figures 4A–D** shows the risk curve and plot of the training and testing cohort patients, with an increase in mortality associated with increased risk scores. **Figures 4E, F** show the heatmap of eight cuproptosis-related lncRNAs expression profiles for every patient in the training and testing cohorts. Taken together, these findings demonstrated that the risk score is a good survival outcome predictor in HNSCC. The survival curves for both cohorts demonstrated that the low-risk HNSCC patients had higher OS (**Figures 4G, H**).

### Verification of the cuproptosis-related LncRNAs prognostic risk model accuracy

**Figure 5:**
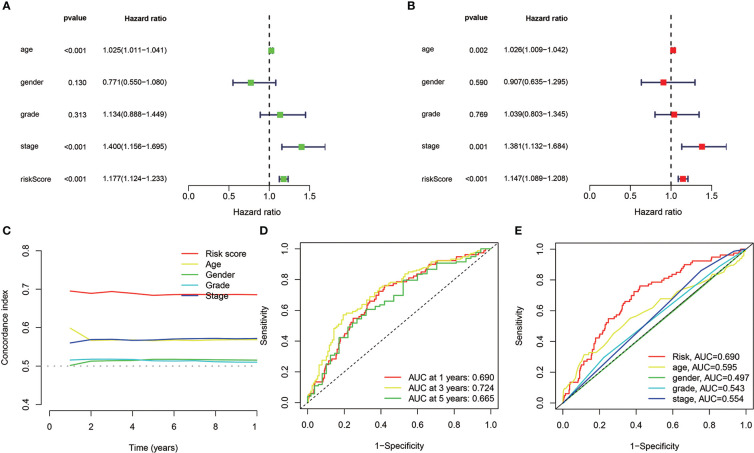
In TCGA cohorts, the cuproptosis-related lncRNAs prognostic risk model assessment and the HNSCC clinical features. **(A, B)** The clinical characteristics and risk score with the OS univariate and multivariate analyses. **(C)** Risk score and clinical properties of concordance indexes. **(D)** The 1-, 3-, and 5-year OS ROC curves. **(E)** The clinical characteristics and risk score ROC curves.

Univariate/multivariate Cox regression was performed to determine if the eight cuproptosis-related lncRNAs had substantial predictive value for HNSCC patients (HRs were 1.177, 95% CI: 1.124–1.233, *p*< 0.001 and 1.147, 95% CI: 1.089–1.208, *p*< 0.001, respectively) as shown in **Figures 5A, B**, indicating that age and stage were associated with the risk profile of the eight cuproptosis**-**related lncRNAs. The risk score concordance index was always larger than any other clinical component over time, implying that the risk grade may be a more accurate predictor of HNSCC outcomes (**Figure 5C**). AUC values of 1-, 3-, and 5-year survival outcomes were 0.690, 0.78524, and 0.665, respectively (**Figure 5D**). Moreover, the risk grade AUC was greater than the other clinicopathological variables, such as age, gender, stage, as well as grade, indicating that eight cuproptosis-related lncRNAs predictive model for HNSCC was reliable (**Figure 5E**).

### Cuproptosis-related lncRNAs prognostic nomogram development and evaluation

**Figure 6:**
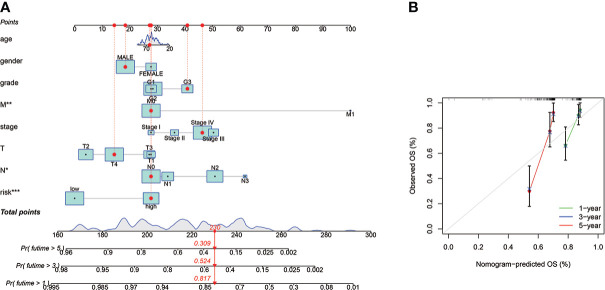
The prognostic nomogram construction and evaluation. **(A)** The nomogram was established for HNSCC patients’ OS prediction at 1, 2, and 3 years, a personalized OS prediction model was made according to gender, age, grade, risk score, TMN, and stage. The nomogram was made for the 1-, 3-, and 5-year OS prediction by the risk levels and variables. **(B)** The nomogram calibration plot for OS prediction at 1, 2, and 3 years, The nomogram prediction accuracy was confirmed by calibration plots highly congruent with the actual 1- and 3-year OS values. *means p < 0.05, **means p < 0.01, ***means p < 0.0001.

For individuals with HNSCC, a personalized OS prediction model was made according to gender, age, grade, risk score, TMN, and stage. The nomogram was made for the 1-, 3-, and 5-year OS prediction by the risk levels and variables. The nomogram prediction accuracy was confirmed by calibration plots highly congruent with the actual 1- and 3-year OS values (**Figures 6A, B**).

### PCA verified the cuproptosis-related lncRNAs model grouping ability

**Figure 7:**
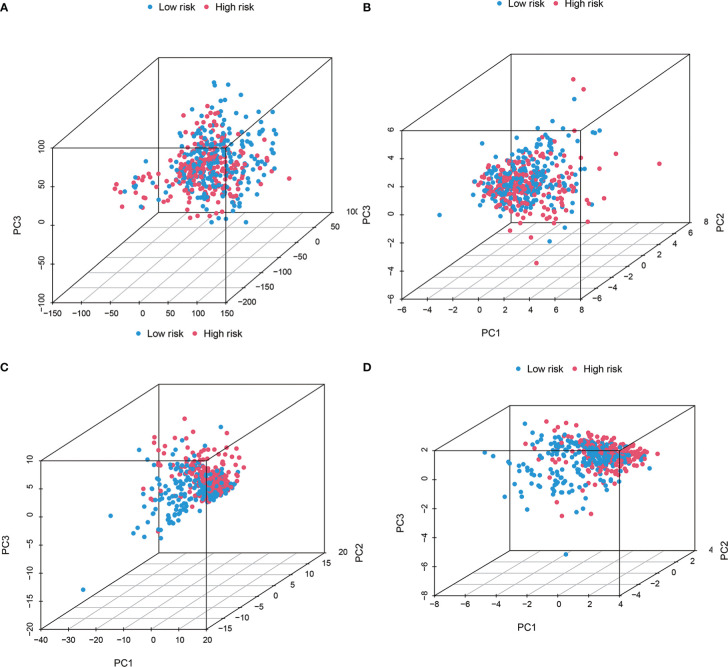
The Low-risk and high-risk groups displayed different stemness statuses. The two risk groups’ principal component analysis on the basis of **(A)** entire gene expression profiles, **(B)** 19 cuproptosis genes, **(C)** 8 cuproptosis-related lncRNAs, as well as **(D)** risk model constructed by eight cuproptosis-related lncRNAs representation profiles in TCGA cohorts. The results showed that the low-risk and high-risk groups based on the cuproptosis-related lncRNAs and risk model were distributed in distinct directions.

PCA results revealed different entire gene expression profile patterns (**Figure 7A**) related to eight cuproptosis-lncRNAs patterns (**Figure 7B**), 19 cuproptosis gene patterns (**Figure 7C**), and a risk model depending on the eight cuproptosis-related lncRNAs representation profiles in the TCGA dataset (**Figures 7D**), indicating that the developed prognostic model could significantly differentiate between the two risk groups.

### TMB, TIME, and cancer immunotherapy response estimation using cuproptosis-related lncRNA model

**Figure 8:**
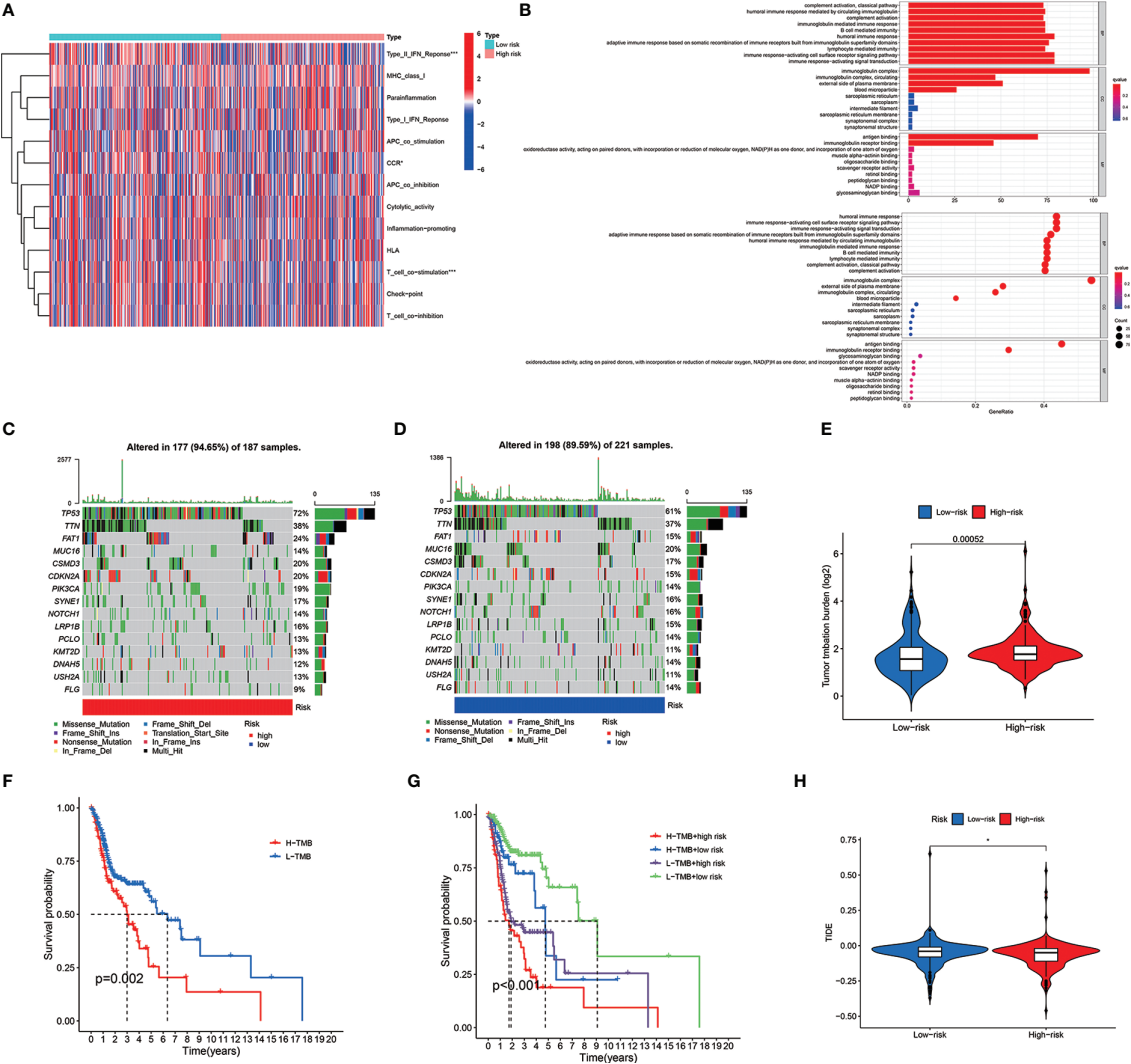
Tumor immune microenvironment and cancer immunotherapy response estimation by cuproptosis-related lncRNA model in TCGA entire set. **(A)** Every patient standard immunity index. **(B)** GO enrichment analysis. **(C, D)** Gene mutation information displayed in Waterfall plot and the high-risk group has higher mutation frequencies compared to the low-risk group. **(E)** TMB difference between the two risk groups. **(F)** OS Kaplan-Meier curve analysis displayed for patients who rely on TMB. **(G)** OS Kaplan-Meier curve analysis according to TMB and risk score **(H)** TIDE prediction differences in both groups. *means p < 0.05, ***means p < 0.0001.

Several immune cells, functions, and pathways enrichment levels and activity were analyzed according to the cuproptosis-related lncRNAs model in 482 HNSCC samples. The results revealed that the two risk groups have considerable differences in the immune indicators expression (**Figure 8A**). GO enrichment analysis, which represented the GO terms for several biological processes related to the immune system, was performed to explore the underlying molecular mechanisms (**Figure 8B**). The maftools R package was utilized for the mutation data analysis, classifying the mutations according to the variant effect predictor. **Figures 8C. D** reveal the top 20 driver genes with the largest mutation frequency in the two risk groups. The TMB scores were then computed according to the TCGA somatic mutation data, with the high-risk group having higher TMB scores, indicating a tight correlation between the cuproptosis-based classifier index and TMB (**Figure 8E**). The higher the TMB score, the poorer the prognosis in terms of survival outcomes (**Figure 8F**) (*p* = 0.002). Furthermore, high and low TMB scores in low-risk groups correlated with a better prognosis (**Figure 8G**). Next, the cuproptosis-related lncRNA model correlations were revealed. We also investigated immunotherapeutic biomarkers and concluded that high-risk group exhibited high response to immunotherapy; therefore, this cuproptosis-based classifier index could indicate the TIDE prediction (**Figure 8H**).

### Investigation of the immune factors and clinical treatment

The GSEA software was utilized for investigating the two groups’ biological function differences to explore the high-risk group in the Kyoto Encyclopedia of Genes and Genomes (KEGG) pathway in the entire set. The pathways were correlated with immunity, such as the “T cell receptor signaling pathway”, “Fc epsilon RI-mediated signaling”, and “VEGF signaling pathway” (all |NES| >1.5; FDR<0.25; *p*<0.05) (**Figure S1A**). From the immune cell bubble chart, high number of immune cells showed a link to high-risk group on various platforms and in the document, comprising T cell CD8+, T cell CD4+, B cell at TIMER, Myeloid dendritic cell activated, T cell CD4+ naïve, B cell at XCELL, Macrophage M2, B cell, Monocyte at QUANTISEQ, and T cell CD8+, B cell, and Endothelial cell at EPIC (all *p*< 0.05) (**Figure S1B**). Also, T cell CD4+ naive_CIBERSORT, Eosinophil_CIBERSORT, and Macrophage M0_CIBERSORT specifically were positively linked to the risk score, whereas B cell memory_XCELL, B cell plasma_CIBERSORT-ABS, Endothelial cell_XCELL, and Macrophage M2_CIBERSORT-ABS were negatively linked to the risk score (**Figure S1C**). In the low-risk group, most immunological checkpoints were more activated (**Figure S1D**), with more immune cells and higher immune function scores (**Figures S1E, F**). Therefore, the ideal checkpoint agonist for HNSCC patients could be selected based on their risk mode. The IC50 of 10 immunotherapeutic medicines, including Bexarotene, Bleomycin, and Gemcitabine, was lower in the high-risk group (**Figure S1G**).

### Validation of the cuproptosis-associated lncRNAs in HNSCC tissues

**Figure 9:**
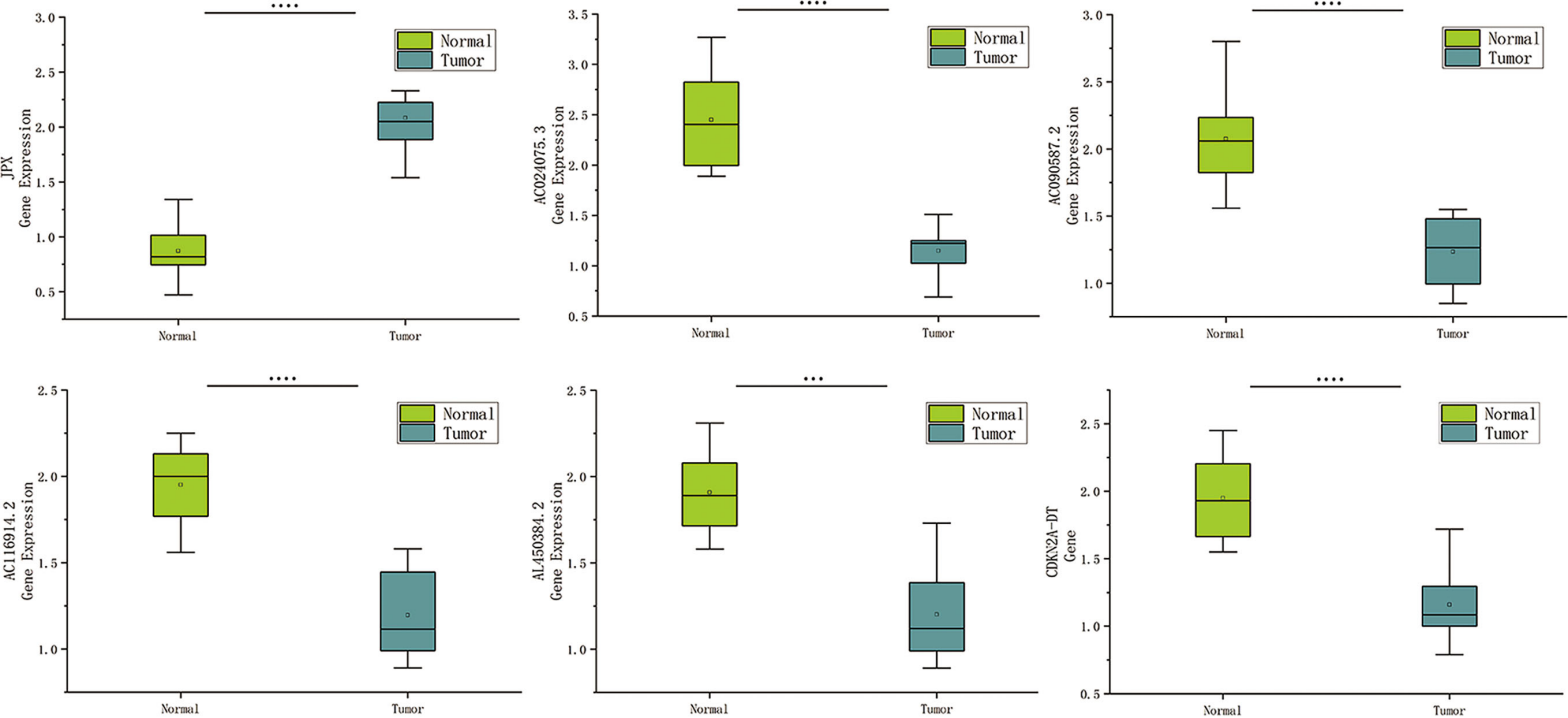
Expression of cuproptosis-related lncRNAs in HNSCC patients. Relative of RNA expression of six cuproptosis-related lncRNAs between cancerous and adjacent normal tissues. ***means p < 0.0001, ****means p < 0.0001.

Based on human paired HNSCC tissues obtained by surgery, we validated the differential expression of six risk lncRNAs included in the risk model by qRT-PCR assays. The differential analysis revealed that JPX was significantly upregulated in HNSCC tissues, while AC024075.3, AC090587.2, AC116914.2, AL450384.2, CDKN2A-DT were downregulated in HNSCC tissues (**Figure 9**).

## Discussion

HNSCC is regarded as a heterogeneous group of carcinomas composing several distinct biological and clinical entities that determine the prognosis and curative effect of HNSCC individuals (2, 3). It has been proposed that identifying biomarkers from database mining is feasible to predict HNSCC prognosis (24, 25). Recently, increasing evidence has indicated that cuproptosis-related genes are important in HNSCC treatment and prognosis. For example, increased NFE2L2 and TMB pathway changes were linked to radiation resistance in laryngeal squamous cell carcinoma (LSCC) (26), and NLRP3 activation could promote carcinogenesis and chemoresistance in HNSCC (27, 28), and ATP7B upregulation promoted chemoresistance of HNSCC (29). The ferroptosis-related lncRNAs signature could also predict HNSCC prognosis (30, 31), but there were no reports of the cuproptosis-related lncRNA signature predictive potential or therapeutic value for HNSCC.

In this study, eight cuproptosis-related lncRNAs were selected as a novel prognostic signature, AC024075.3, AC090587.2, AC116914.2, AL450384.2, CDKN2A-DT, FAM27E3, JPX, and LNC01089. To the best of our knowledge, these cuproptosis-related prognostic lncRNAs were first established in HNSCC by our study. Several of these lncRNAs have been investigated; for example, AC090587.2 can act as an m6A-related lncRNA with great prognostic power in HNSCC (25). Several new studies related the AC116914.2 role in HNSCC to its hypoxia and autophagy potential, which plays a pivotal role in the prognostic power (32, 33). As reported, JPX has a pivotal function in the development of several cancers, such as colorectal (34), gastric (35), cervical (36), and lung cancers (37). The function of the other five lncRNAs has not yet been reported, so future research studies will require molecular investigations to explore those functions and mechanisms in HNSCC.

According to the risk scores of the established model, low-risk patients survived longer and had a better prognosis. The ROC curve demonstrated that the signature was reliable and stable in terms of satisfactory predictive performance. The stage, age, and risk score were also three predictive factors, demonstrating that the developed signature could predict the HNSCC prognosis as an independent prognostic factor.

Traditional strategies have provided disappointing therapeutic results; thus, immunotherapy has become a promising tool for HNSCC treatment (38). However, only a small population of HNSCC patients benefit from these costly therapies (38) because the TIME consisting of immune cells, signaling molecules, blood vessels, and the extracellular matrix (ECM) is a complex and dynamic environment (39). Accumulating evidence has demonstrated the importance of lncRNAs as regulators in the TIME (40). As expected, the two groups had a different TIME, showing notable differences in immune indicator expression, such as Type_II_IFN_Response, CCR, and T_cell_co-stimulation. For the high-risk group, the underlying mechanisms of the poor prognosis were revealed by GO enrichment analysis.

Currently, only PD1 and PD-L1 have been validated as predictive biomarkers of immune checkpoint inhibitor response in HNSCC (41). In this study, the low-risk group had more immune cells and higher immune function scores, with better activation of most immune checkpoints such as BTLA, CD28, and CD27. Recently, regarding the response to PD-L1 treatment, it has been reported that the TMB could be a reliable biomarker (42). The cuproptosis-based classifier index and TMB had a strong linkage; the higher the TMB, the worse the prognosis. Furthermore, it implied that this cuproptosis-based classifier index could be an indicator for TIDE prediction. The high-risk patients had a greater response to immunotherapy; therefore, the established model can provide novel and reliable immune biomarkers for treating HNSCC.

As well as the cytotoxic anti-cancer agents and epidermal growth factor receptor tyrosine kinase inhibitors, two anti-PD-1 antibodies (pembrolizumab and nivolumab) are currently utilized in recurrent or metastatic HNSCC patients, yet with limited benefits (38). Based on the IC50 difference of various drugs in different HNSCC clusters, we found several personalized potentiate precise medications, such as Bexarotene, Bleomycin, and Gemcitabine, but this precision and personalized medicine require further study.

Overall, this study suggests that the cuproptosis-related lncRNA prognostic signature could bring a breakthrough in clinical practice for HNSCC patients, providing new information about the patients with different immunophenotype stratification and insight into the immune molecular mechanisms of HNSCC. However, this study has some limitations. First, HNSCC encompasses several types of cancers, so separate, detailed analyses should consider the HNSCC subtypes. Second, the results based on a public database need further validation on other data sets, especially the lncRNA signature, and larger study samples are required for verification. Finally, further studies are required to better understand the molecular mechanisms and potential treatment targets of HNSCC.

## Data availability statement

The datasets presented in this study can be found in online repositories. The names of the repository/repositories and accession number(s) can be found in the article/**Supplementary material**.

## Author contributions

Study design and concept: JZ and SW. Data acquisition: LZ, YH, and HT. Data analysis and interpretation: LZ, YH, and HT. Manuscript preparation: LZ, YH, HT, and XL. Manuscript review: JZ, SW, and QC. The final manuscript was approved by all authors.

## Supplementary material

Supplementary Figure 1 **(A)** The top nine pathways GSEA showed excellent enrichment in both groups **(B)** The risk groups immune cell bubble, high number of immune cells showed a link to high-risk group on various platforms. **(C)** The risk score and immune cells correlation, T cell CD4+ naive_CIBERSORT, Eosinophil_CIBERSORT, and Macrophage M0_CIBERSORT specifically were positively linked to the risk score, whereas B cell memory_XCELL, B cell plasma_CIBERSORT-ABS, Endothelial cell_XCELL, and Macrophage M2_CIBERSORT-ABS were negatively linked to the risk score. **(D)** The checkpoints expression difference in both groups, most immunological checkpoints were more activated. **(E)** The risk groups immunotherapy prediction, The IC50 of 10 immunotherapeutic medicines, including Bexarotene, Bleomycin, and Gemcitabine, was lower in the high-risk group.

Supplementary information 2 R packages of Uni-and multi-variate Cox regression analyses, KM survival analysis, PCA, ROC, Nomogram, GSEA, chemosensitivity prediction model.
