# Supplementary figures and images for: Cuproptosis-related LncRNAs are potential prognostic and immune response markers for patients with HNSCC via the integration of bioinformatics analysis and experimental validation
Source: Front Oncol. 2022 Dec 22;12:1030802. doi: 10.3389/fonc.2022.1030802 (PMC9815527; doi:10.3389/fonc.2022.1030802)

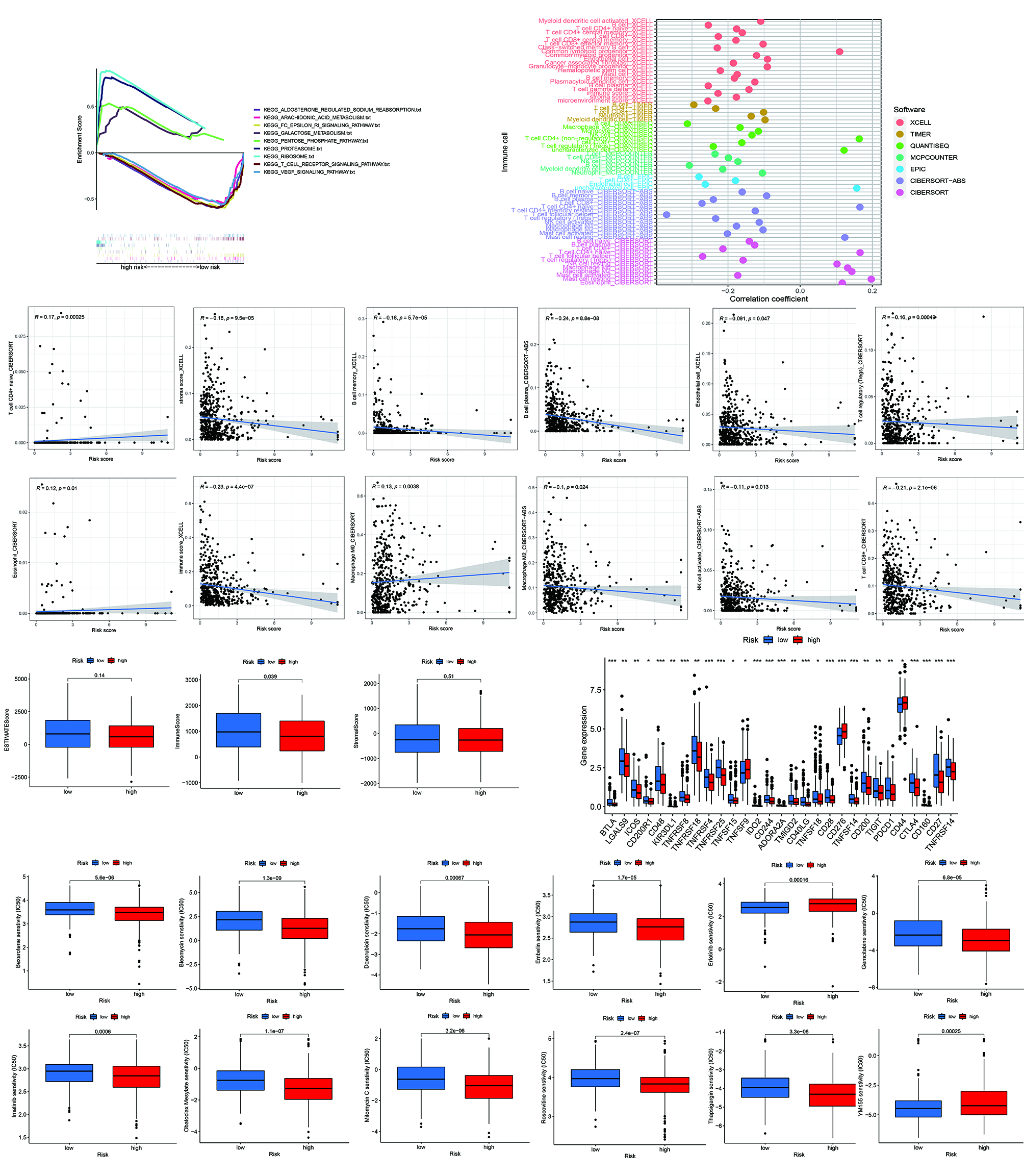

Supplement: Supplementary Figure 1 — (A) The top nine pathways GSEA showed excellent enrichment in both groups (B) The risk groups immune cell bubble, high number of immune cells showed a link to high-risk group on various platforms. (C) The risk score and immune cells correlation, T cell CD4+ naive_CIBERSORT, Eosinophil_CIBERSORT, and Macrophage M0_CIBERSORT specifically were positively linked to the risk score, whereas B cell memory_XCELL, B cell plasma_CIBERSORT-ABS, Endothelial cell_XCELL, and Macrophage M2_CIBERSORT-ABS were negatively linked to the risk score. (D) The checkpoints expression difference in both groups, most immunological checkpoints were more activated. (E) The risk groups immunotherapy prediction, The IC50 of 10 immunotherapeutic medicines, including Bexarotene, Bleomycin, and Gemcitabine, was lower in the high-risk group. [file Image_1.tif]
