# Supplementary material for: Cuproptosis-related LncRNAs are potential prognostic and immune response markers for patients with HNSCC via the integration of bioinformatics analysis and experimental validation
Source: Front Oncol. 2022 Dec 22;12:1030802. doi: 10.3389/fonc.2022.1030802 (PMC9815527; doi:10.3389/fonc.2022.1030802)
Supplement: Supplementary information 2 — R packages of Uni-and multi-variate Cox regression analyses, KM survival analysis, PCA, ROC, Nomogram, GSEA, chemosensitivity prediction model. [file DataSheet_1.pdf]

1. survial.R

```
#install.packages("survival")
#install.packages("survminer")


#引用包
library(survival)
library(survminer)
setwd("C:\\biowolf\\cuproptosis\\17.survival")        #设置工作目录

#定义生存分析的函数
bioSurvival=function(inputFile=null, outFile=null){
	#读取输入文件
	rt=read.table(inputFile, header=T, sep="\t")
	#比较高低风险组生存差异，得到显著性 p 值
	diff=survdiff(Surv(futime, fustat) ~risk,data = rt)
	pValue=1-pchisq(diff$chisq,df=1)
	if(pValue<0.001){
		pValue="p<0.001"
	}else{
		pValue=paste0("p=",sprintf("%.03f",pValue))
	}
	fit <- survfit(Surv(futime, fustat) ~ risk, data = rt)

	#绘制生存曲线
	surPlot=ggsurvplot(fit,
		           data=rt,
		           conf.int=F,
		           pval=pValue,
		           pval.size=6,
		           legend.title="Risk",
		           legend.labs=c("High risk", "Low risk"),
		           xlab="Time(years)",
		           ylab="Overall survival",
		           break.time.by = 1,
		           palette=c("red", "blue"),
		           risk.table=TRUE,
		           risk.table.title="",
		           risk.table.col = "strata",
		           risk.table.height=.25)

	#输出图形
```

```
    pdf(file=outFile, width = 6.5, height =5.5, onefile = FALSE)
    print(surPlot)
    dev.off()
}

#调用函数，绘制生存曲线
bioSurvival(inputFile="risk.train.txt", outFile="surv.train.pdf")
bioSurvival(inputFile="risk.test.txt", outFile="surv.test.pdf")
bioSurvival(inputFile="risk.all.txt", outFile="surv.all.pdf")
```

2. riskPlot

```
#install.packages("pheatmap")


library(pheatmap)                #引用包
setwd("C:\\biowolf\\cuproptosis\\19.riskPlot")            #设置工作目录

#定义风险曲线的函数
bioRiskPlot=function(inputFile=null, project=null){
 rt=read.table(inputFile, header=T, sep="\t", check.names=F, row.names=1)        #读取输入文件
 rt=rt[order(rt$riskScore),]            #按照病人风险打分对样品进行排序

 #绘制风险曲线
 riskClass=rt[,"risk"]
 lowLength=length(riskClass[riskClass=="low"])
 highLength=length(riskClass[riskClass=="high"])
 lowMax=max(rt$riskScore[riskClass=="low"])
 line=rt[,"riskScore"]
 line[line>10]=10
 pdf(file=paste0(project, ".riskScore.pdf"), width=7, height=4)
 plot(line, type="p", pch=20,
         xlab="Patients (increasing risk socre)",
         ylab="Risk score",
         col=c(rep("blue",lowLength),rep("red",highLength)) )
 abline(h=lowMax,v=lowLength,lty=2)
 legend("topleft", c("High risk","Low Risk"),bty="n",pch=19,col=c("red","blue"),cex=1.2)
 dev.off()

 #绘制生存状态图
```

```
 color=as.vector(rt$fustat)
 color[color==1]="red"
 color[color==0]="blue"
 pdf(file=paste0(project, ".survStat.pdf"), width=7, height=4)
 plot(rt$futime, pch=19,
          xlab="Patients (increasing risk socre)",
          ylab="Survival time (years)",
          col=color)
 legend("topleft", c("Dead","Alive"),bty="n",pch=19,col=c("red","blue"),cex=1.2)
 abline(v=lowLength,lty=2)
 dev.off()

 #定义热图注释的颜色
 ann_colors=list()
 bioCol=c("blue", "red")
 names(bioCol)=c("low", "high")
 ann_colors[["Risk"]]=bioCol

 #绘制风险热图
 rt1=rt[c(3:(ncol(rt)-2))]
 rt1=t(rt1)
 annotation=data.frame(Risk=rt[,ncol(rt)])
 rownames(annotation)=rownames(rt)
 pdf(file=paste0(project, ".heatmap.pdf"), width=7, height=4)
 pheatmap(rt1,
                annotation=annotation,
                annotation_colors = ann_colors,
                cluster_cols = FALSE,
                cluster_rows = FALSE,
                show_colnames = F,
                scale="row",
                color = colorRampPalette(c(rep("blue",3.5), "white", rep("red",3.5)))(50),
                fontsize_col=3,
                fontsize=7,
                fontsize_row=8)
 dev.off()
}

#tarin 组风险曲线
bioRiskPlot(inputFile="risk.train.txt", project="train")
#test 组风险曲线
bioRiskPlot(inputFile="risk.test.txt", project="test")
#所有样品风险曲线
bioRiskPlot(inputFile="risk.all.txt", project="all")
```

3. ROC

```
#install.packages("survival")
#install.packages("survminer")
#install.packages("timeROC")


#引用包
library(survival)
library(survminer)
library(timeROC)

riskFile="risk.all.txt"       #风险文件
cliFile="clinical.txt"        #临床数据文件
setwd("C:\\biowolf\\cuproptosis\\21.ROC")      #修改工作目录

#读取风险输入文件
risk=read.table(riskFile, header=T, sep="\t", check.names=F, row.names=1)
risk=risk[,c("futime", "fustat", "riskScore")]

#读取临床数据文件
cli=read.table(cliFile, header=T, sep="\t", check.names=F, row.names=1)

#合并数据
samSample=intersect(row.names(risk), row.names(cli))
risk1=risk[samSample,,drop=F]
cli=cli[samSample,,drop=F]
rt=cbind(risk1, cli)

#定义颜色
bioCol=rainbow(ncol(rt)-1, s=0.9, v=0.9)


######绘制 1 3 5 年的 ROC 曲线######
ROC_rt=timeROC(T=risk$futime,delta=risk$fustat,
               marker=risk$riskScore,cause=1,
               weighting='aalen',
               times=c(1,3,5),ROC=TRUE)
pdf(file="ROC.pdf", width=5, height=5)
plot(ROC_rt,time=1,col=bioCol[1],title=FALSE,lwd=2)
plot(ROC_rt,time=3,col=bioCol[2],add=TRUE,title=FALSE,lwd=2)
plot(ROC_rt,time=5,col=bioCol[3],add=TRUE,title=FALSE,lwd=2)
legend('bottomright',
```

```
       c(paste0('AUC at 1 years: ',sprintf("%.03f",ROC_rt$AUC[1])),
          paste0('AUC at 3 years: ',sprintf("%.03f",ROC_rt$AUC[2])),
          paste0('AUC at 5 years: ',sprintf("%.03f",ROC_rt$AUC[3]))),
       col=bioCol[1:3], lwd=2, bty = 'n')
dev.off()


######绘制临床的 ROC 曲线######
predictTime=1        #定义预测年限
aucText=c()
pdf(file="cliROC.pdf", width=5, height=5)
#绘制风险得分的 ROC 曲线
i=3
ROC_rt=timeROC(T=risk$futime,
               delta=risk$fustat,
               marker=risk$riskScore, cause=1,
               weighting='aalen',
               times=c(predictTime),ROC=TRUE)
plot(ROC_rt, time=predictTime, col=bioCol[i-2], title=FALSE, lwd=2)
aucText=c(paste0("Risk", ", AUC=", sprintf("%.3f",ROC_rt$AUC[2])))
abline(0,1)
#对临床数据进行循环，绘制临床数据的 ROC 曲线
for(i in 4:ncol(rt)){
 ROC_rt=timeROC(T=rt$futime,
                delta=rt$fustat,
                marker=rt[,i], cause=1,
                weighting='aalen',
                times=c(predictTime),ROC=TRUE)
 plot(ROC_rt, time=predictTime, col=bioCol[i-2], title=FALSE, lwd=2, add=TRUE)
 aucText=c(aucText, paste0(colnames(rt)[i],", AUC=",sprintf("%.3f",ROC_rt$AUC[2])))
}
#绘制图例，得到 ROC 曲线下的面积
legend("bottomright", aucText,lwd=2,bty="n",col=bioCol[1:(ncol(rt)-1)])
dev.off()
```

4. NOMO

```
#install.packages("survival")
#install.packages("regplot")
#install.packages("rms")


#引用包
```

```
library(survival)
library(regplot)
library(rms)

riskFile="risk.all.txt"           #风险文件
cliFile="clinical.txt"            #临床数据文件
setwd("C:\\biowolf\\cuproptosis\\23.Nomo")         #修改工作目录

#读取风险输入文件
risk=read.table(riskFile, header=T, sep="\t", check.names=F, row.names=1)

#读取临床数据文件
cli=read.table(cliFile, header=T, sep="\t", check.names=F, row.names=1)
cli=cli[apply(cli,1,function(x)any(is.na(match('unknow',x)))),,drop=F]
cli$Age=as.numeric(cli$Age)

#合并数据
samSample=intersect(row.names(risk), row.names(cli))
risk1=risk[samSample,,drop=F]
cli=cli[samSample,,drop=F]
rt=cbind(risk1[,c("futime", "fustat", "risk")], cli)

#绘制列线图
res.cox=coxph(Surv(futime, fustat) ~ . , data = rt)
nom1=regplot(res.cox,
              plots = c("density", "boxes"),
              clickable=F,
              title="",
              points=TRUE,
              droplines=TRUE,
              observation=rt[20,],
              rank="sd",
              failtime = c(1,3,5),
              prfail = F)

#列线图风险打分
nomoRisk=predict(res.cox, data=rt, type="risk")
rt=cbind(risk1, Nomogram=nomoRisk)
outTab=rbind(ID=colnames(rt), rt)
write.table(outTab, file="nomoRisk.txt", sep="\t", col.names=F, quote=F)

#校准曲线
pdf(file="calibration.pdf", width=5, height=5)
#1 年校准曲线
```

```
f <- cph(Surv(futime, fustat) ~ Nomogram, x=T, y=T, surv=T, data=rt, time.inc=1)
cal <- calibrate(f, cmethod="KM", method="boot", u=1, m=(nrow(rt)/3), B=1000)
plot(cal, xlim=c(0,1), ylim=c(0,1),
  xlab="Nomogram-predicted OS (%)", ylab="Observed OS (%)", lwd=1.5, col="green", sub=F)
#3 年校准曲线
f <- cph(Surv(futime, fustat) ~ Nomogram, x=T, y=T, surv=T, data=rt, time.inc=3)
cal <- calibrate(f, cmethod="KM", method="boot", u=3, m=(nrow(rt)/3), B=1000)
plot(cal, xlim=c(0,1), ylim=c(0,1), xlab="", ylab="", lwd=1.5, col="blue", sub=F, add=T)
#5 年校准曲线
f <- cph(Surv(futime, fustat) ~ Nomogram, x=T, y=T, surv=T, data=rt, time.inc=5)
cal <- calibrate(f, cmethod="KM", method="boot", u=5, m=(nrow(rt)/3), B=1000)
plot(cal, xlim=c(0,1), ylim=c(0,1), xlab="", ylab="",  lwd=1.5, col="red", sub=F, add=T)
legend('bottomright', c('1-year', '3-year', '5-year'),
    col=c("green","blue","red"), lwd=1.5, bty = 'n')
dev.off()
```

5. PCA

```
#if (!requireNamespace("BiocManager", quietly = TRUE))
#     install.packages("BiocManager")
#BiocManager::install("limma")

#install.packages("scatterplot3d")


#引用包
library(limma)
library(scatterplot3d)
setwd("C:\\biowolf\\cuproptosis\\25.PCA")          #设置工作目录

#定义 PCA 分析函数
myPCA=function(input=null,output=null){
 #读取表达数据文件
 rt=read.table(input, header=T, sep="\t", check.names=F)
 rt=as.matrix(rt)
 rownames(rt)=rt[,1]
 exp=rt[,2:ncol(rt)]
 dimnames=list(rownames(exp),colnames(exp))
 data=matrix(as.numeric(as.matrix(exp)),nrow=nrow(exp),dimnames=dimnames)
 data=avereps(data)
 data=data[rowMeans(data)>0.5,]
```

```
 #删除正常样品
 type=sapply(strsplit(colnames(data),"\\-"),"[",4)
 type=sapply(strsplit(type,""),"[",1)
 type=gsub("2","1",type)
 data=t(data[,type==0])
 rownames(data)=gsub("(.*?)\\-(.*?)\\-(.*?)\\-(.*?)\\-.*","\\1\\-\\2\\-\\3",rownames(data))

 #读取 risk 风险文件
 risk=read.table("risk.all.txt", header=T, sep="\t", row.names=1, check.names=F)
 sameSample=intersect(rownames(data),rownames(risk))
 data=data[sameSample,]
 risk=risk[sameSample,]
 group=as.vector(risk[,"risk"])

 #PCA 分析
 data.class <- rownames(data)
 data.pca <- prcomp(data, scale. = TRUE)

 #绘制 PCA 图形
 color=ifelse(group=="low",4,2)
 pcaPredict=predict(data.pca)
 pdf(file=output, width=7, height=7)
 par(oma=c(1,1,2.5,1))
 s3d=scatterplot3d(pcaPredict[,1:3], pch = 16, color=color, angle=35)
 legend("top", legend = c("Low risk","High risk"),pch = 16, inset = -0.2, box.col="white", xpd = TRUE, horiz = TRUE,col=c(4,2))
 dev.off()
}

######绘制所有基因的 PCA 图，将 04 节课 symbol.txt 复制到当前目录
myPCA(input="symbol.txt",output="PCA.allGene.pdf")
######绘制铜死亡相关基因的 PCA 图，将 09 节课 cuproptosisExp.txt 复制到当前目录
myPCA(input="cuproptosisExp.txt",output="PCA.cuproptosisGene.pdf")
######绘制铜死亡相关 lncRNA 的 PCA 图，将 09 节课 cuproptosisLncExp.txt 复制到当前目录
myPCA(input="cuproptosisLncExp.txt",output="PCA.cuproptosisLncRNA.pdf")


######读取风险文件,绘制模型 lncRNA 的 PCA 图，将 14 节课 risk.all.txt 复制到当前目录
risk=read.table("risk.all.txt", header=T, sep="\t", check.names=F, row.names=1)
```

```
data=risk[,3:(ncol(risk)-2)]
group=as.vector(risk[,"risk"])

#PCA 分析
data.class <- rownames(data)
data.pca <- prcomp(data, scale. = TRUE)

#可视化
color=ifelse(group=="low",4,2)
pcaPredict=predict(data.pca)
pdf(file="PCA.riskLnc.pdf", width=6.5, height=6)
par(oma=c(1,1,2.5,1))
s3d=scatterplot3d(pcaPredict[,1:3], pch = 16, color=color, angle=35)
legend("top", legend = c("Low risk","High risk"),pch = 16, inset = -0.2, box.col="white", xpd = TRUE, horiz = TRUE,col=c(4,2))
dev.off()
```

6. KEGG

```
#install.packages("colorspace")
#install.packages("stringi")
#install.packages("ggplot2")
#install.packages("circlize")
#install.packages("RColorBrewer")

#if (!requireNamespace("BiocManager", quietly = TRUE))
#    install.packages("BiocManager")
#BiocManager::install("org.Hs.eg.db")
#BiocManager::install("DOSE")
#BiocManager::install("clusterProfiler")
#BiocManager::install("enrichplot")
#BiocManager::install("ComplexHeatmap")


#引用包
library(clusterProfiler)
library(org.Hs.eg.db)
library(enrichplot)
library(ggplot2)
library(circlize)
library(RColorBrewer)
```

```
library(dplyr)
library(ComplexHeatmap)

pvalueFilter=0.05          #p 值过滤条件
qvalueFilter=0.05          #矫正后的 p 值过滤条件

#定义颜色
colorSel="qvalue"
if(qvalueFilter>0.05){
 colorSel="pvalue"
}

setwd("C:\\biowolf\\cuproptosis\\28.KEGG")                  #设置工作目录
rt=read.table("riskDiff.txt", header=T, sep="\t", check.names=F)          #读取输入文件

#提取差异基因的名称,将基因名字转换为基因 id
genes=unique(as.vector(rt[,1]))
entrezIDs=mget(genes, org.Hs.egSYMBOL2EG, ifnotfound=NA)
entrezIDs=as.character(entrezIDs)
rt=data.frame(genes, entrezID=entrezIDs)
gene=entrezIDs[entrezIDs!="NA"]             #去除基因 id 为 NA 的基因
#gene=gsub("c\\(\"(\\d+)\".*", "\\1", gene)

#KEGG 富集分析
kk <- enrichKEGG(gene=gene, organism="hsa", pvalueCutoff=1, qvalueCutoff=1)
KEGG=as.data.frame(kk)
KEGG$geneID=as.character(sapply(KEGG$geneID,function(x)paste(rt$genes[match(strsplit(x,"/")[[1]],as.character(rt$entrezID))],collapse="/")))
KEGG=KEGG[(KEGG$pvalue<pvalueFilter & KEGG$qvalue<qvalueFilter),]
#保存显著富集的结果
write.table(KEGG, file="KEGG.txt", sep="\t", quote=F, row.names = F)

#定义显示通路的数目
showNum=30
if(nrow(KEGG)<showNum){
 showNum=nrow(KEGG)
}

#柱状图
pdf(file="barplot.pdf", width=9, height=7)
barplot(kk, drop=TRUE, showCategory=showNum, label_format=130, color=colorSel)
dev.off()
```

```
#气泡图
pdf(file="bubble.pdf", width = 9, height = 7)
dotplot(kk, showCategory=showNum, orderBy="GeneRatio", label_format=130, color=colorSel)
dev.off()

###########绘制 KEGG 圈图###########
Pathway.col=c("#90EE90", "#E7B800", "#00AFBB")
showNum=18
data=KEGG[order(KEGG$p.adjust),]
if(nrow(KEGG)>showNum){
 data=data[1:showNum,]
}
data$Pathway="KEGG"
main.col = Pathway.col[as.numeric(as.factor(data$Pathway))]

#整理圈图数据
BgGene = as.numeric(sapply(strsplit(data$BgRatio,"/"),'[',1))
Gene = as.numeric(sapply(strsplit(data$GeneRatio,'/'),'[',1))
ratio = Gene/BgGene
logpvalue = -log(data$pvalue,10)
logpvalue.col = brewer.pal(n = 8, name = "Reds")
f = colorRamp2(breaks = c(0,2,4,6,8,10,15,20), colors = logpvalue.col)
BgGene.col = f(logpvalue)
df = data.frame(KEGG=data$ID,start=1,end=max(BgGene))
rownames(df) = df$KEGG
bed2 = data.frame(KEGG=data$ID,start=1,end=BgGene,BgGene=BgGene,BgGene.col=BgGene.col)
bed3 = data.frame(KEGG=data$ID,start=1,end=Gene,BgGene=Gene)
bed4 = data.frame(KEGG=data$ID,start=1,end=max(BgGene),ratio=ratio,col=main.col)
bed4$ratio = bed4$ratio/max(bed4$ratio)*9.5

#绘制圈图主体部分
pdf(file="KEGG.circlize.pdf",width=10,height=10)
par(omi=c(0.1,0.1,0.1,1.5))
circos.par(track.margin=c(0.01,0.01))
circos.genomicInitialize(df,plotType="none")
circos.trackPlotRegion(ylim = c(0, 1), panel.fun = function(x, y) {
   sector.index = get.cell.meta.data("sector.index")
   xlim = get.cell.meta.data("xlim")
   ylim = get.cell.meta.data("ylim")
```

```
    circos.text(mean(xlim), mean(ylim), sector.index, cex = 0.8, facing =
"bending.inside", niceFacing = TRUE)
  }, track.height = 0.08, bg.border = NA,bg.col = main.col)

  for(si in get.all.sector.index()) {
    circos.axis(h = "top", labels.cex = 0.6, sector.index = si,track.index = 1,
                major.at=seq(0,max(BgGene),by=100),labels.facing = "clockwise")
  }
  f = colorRamp2(breaks = c(-1, 0, 1), colors = c("green", "black", "red"))
  circos.genomicTrack(bed2, ylim = c(0, 1),track.height = 0.1,bg.border="white",
                      panel.fun = function(region, value, ...) {
                        i = getI(...)
                        circos.genomicRect(region, value, ytop = 0, ybottom = 1,
col = value[,2],
                                           border = NA, ...)
                        circos.genomicText(region, value, y = 0.4, labels = value[,1],
adj=0,cex=0.8,...)
                      })
  circos.genomicTrack(bed3, ylim = c(0, 1),track.height = 0.1,bg.border="white",
                      panel.fun = function(region, value, ...) {
                        i = getI(...)
                        circos.genomicRect(region, value, ytop = 0, ybottom = 1,
col = '#BA55D3',
                                           border = NA, ...)
                        circos.genomicText(region, value, y = 0.4, labels = value[,1],
cex=0.9,adj=0,...)
                      })
  circos.genomicTrack(bed4, ylim = c(0, 10),track.height =
0.35,bg.border="white",bg.col="grey90",
                      panel.fun = function(region, value, ...) {
                        cell.xlim = get.cell.meta.data("cell.xlim")
                        cell.ylim = get.cell.meta.data("cell.ylim")
                        for(j in 1:9) {
                          y = cell.ylim[1] + (cell.ylim[2]-cell.ylim[1])/10*j
                          circos.lines(cell.xlim, c(y, y), col = "#FFFFFF", lwd = 0.3)
                        }
                        circos.genomicRect(region, value, ytop = 0, ybottom =
value[,1], col = value[,2],
                                           border = NA, ...)
                        #circos.genomicText(region, value, y = 0.3, labels =
value[,1], ...)
                      })
  circos.clear()
  #绘制圈图中间的图例
```

```
middle.legend = Legend(
   labels = c('Number of Genes','Number of Select','Rich Factor(0-1)'),
   type="points",pch=c(15,15,17),legend_gp = gpar(col=c('pink','#BA55D3',Pathway.col[1])),
   title="",nrow=3,size= unit(3, "mm")
)
circle_size = unit(1, "snpc")
draw(middle.legend,x=circle_size*0.42)
#绘制 KEGG 分类的图例
main.legend = Legend(
   labels = c("KEGG"),   type="points",pch=15,
   legend_gp = gpar(col=Pathway.col), title_position = "topcenter",
   title = "Pathway", nrow = 3,size = unit(3, "mm"),grid_height = unit(5, "mm"),
   grid_width = unit(5, "mm")
)
#绘制 pvalue 的图例
logp.legend = Legend(
   labels=c('(0,2]','(2,4]','(4,6]','(6,8]','(8,10]','(10,15]','(15,20]','>=20'),
   type="points",pch=16,legend_gp=gpar(col=logpvalue.col),title="-log10(Pvalue)",
   title_position = "topcenter",grid_height = unit(5, "mm"),grid_width = unit(5, "mm"),
   size = unit(3, "mm")
)
lgd = packLegend(main.legend,logp.legend)
circle_size = unit(1, "snpc")
print(circle_size)
draw(lgd, x = circle_size*0.85, y=circle_size*0.55,just = "left")
dev.off()
```

7. .pRRophetic

```
#if (!requireNamespace("BiocManager", quietly = TRUE))
#     install.packages("BiocManager")
#BiocManager::install(c("car", "ridge", "preprocessCore", "genefilter", "sva"))

#install.packages("ggpubr")


#引用包
library(limma)
library(ggpubr)
library(pRRophetic)
library(ggplot2)
```

```
set.seed(12345)

pFilter=0.001                          #pvalue 的过滤条件
expFile="symbol.txt"               #表达数据文件
riskFile="risk.all.txt"          #风险文件
setwd("C:\\biowolf\\cuproptosis\\35.pRRophetic")         #设置工作目录

#获取药物列表
data(cgp2016ExprRma)
data(PANCANCER_IC_Tue_Aug_9_15_28_57_2016)
allDrugs=unique(drugData2016$Drug.name)

#读取表达输入文件,并对数据进行处理
rt = read.table(expFile, header=T, sep="\t", check.names=F)
rt=as.matrix(rt)
rownames(rt)=rt[,1]
exp=rt[,2:ncol(rt)]
dimnames=list(rownames(exp),colnames(exp))
data=matrix(as.numeric(as.matrix(exp)),nrow=nrow(exp),dimnames=dimnames)
data=avereps(data)
data=data[rowMeans(data)>0.5,]

#删掉正常样品
group=sapply(strsplit(colnames(data),"\\-"), "[", 4)
group=sapply(strsplit(group,""), "[", 1)
group=gsub("2","1",group)
data=data[,group==0]
data=t(data)
rownames(data)=gsub("(.*?)\\-(.*?)\\-(.*?)\\-(.*)", "\\1\\-\\2\\-\\3", rownames(data))
data=avereps(data)
data=t(data)

#读取风险输入文件
riskRT=read.table(riskFile, header=T, sep="\t", check.names=F, row.names=1)
riskRT$riskScore[riskRT$riskScore>quantile(riskRT$riskScore,0.99)]=quantile(riskRT$r
iskScore,0.99)

#对药物进行循环
for(drug in allDrugs){
 #预测药物敏感性
 possibleError=tryCatch(
        {senstivity=pRRopheticPredict(data, drug, selection=1, dataset = "cgp2016")},
        error=function(e) e)
       if(inherits(possibleError, "error")){next}
```

```r
senstivity=senstivity[senstivity!="NaN"]
senstivity[senstivity>quantile(senstivity,0.99)]=quantile(senstivity,0.99)

#将风险文件与药物敏感性的结果进行合并
sameSample=intersect(row.names(riskRT), names(senstivity))
risk=riskRT[sameSample, c("riskScore","risk"),drop=F]
senstivity=senstivity[sameSample]
rt=cbind(risk, senstivity)

#设置比较组
rt$risk=factor(rt$risk, levels=c("low", "high"))
type=levels(factor(rt[,"risk"]))
comp=combn(type, 2)
my_comparisons=list()
for(i in 1:ncol(comp)){my_comparisons[[i]]<-comp[,i]}

#获取高低风险组差异的 pvalue
test=wilcox.test(senstivity~risk, data=rt)
diffPvalue=test$p.value
#获取相关性检验的 pvalue
x=as.numeric(rt[,"riskScore"])
y=as.numeric(rt[,"senstivity"])
corT=cor.test(x, y, method="spearman")
corPvalue=corT$p.value

if((diffPvalue<pFilter) & (corPvalue<pFilter)){
    #绘制箱线图
    boxplot=ggboxplot(rt, x="risk", y="senstivity", fill="risk",
                      xlab="Risk",
                      ylab=paste0(drug, " senstivity (IC50)"),
                      legend.title="Risk",
                      palette=c("#0066FF","#FF0000")
                     )+
        stat_compare_means(comparisons=my_comparisons)
    pdf(file=paste0("durgSenstivity.", drug, ".pdf"), width=5, height=4.5)
    print(boxplot)
    dev.off()
    #绘制相关性图形
    df1=as.data.frame(cbind(x,y))
    p1=ggplot(df1, aes(x, y)) +
            xlab("Risk score") + ylab(paste0(drug, " senstivity (IC50)"))+
            geom_point() + geom_smooth(method="lm",formula = y ~ x) +
theme_bw()+
            stat_cor(method = 'spearman', aes(x =x, y =y))
```

```
        #相关性图形
        pdf(file=paste0("Cor.", drug, ".pdf"), width=5, height=4.6)
        print(p1)
        dev.off()
    }
}
```

8, checkpoint

```
#if (!requireNamespace("BiocManager", quietly = TRUE))
#       install.packages("BiocManager")
#BiocManager::install("limma")

#install.packages("ggplot2")
#install.packages("ggpubr")


#引用包
library(limma)
library(reshape2)
library(ggplot2)
library(ggpubr)

expFile="symbol.txt"               #表达数据文件
riskFile="risk.all.txt"            #风险文件
geneFile="gene.txt"                #免疫检查点的基因文件
setwd("C:\\biowolf\\Necroptosis\\30.checkpoint")        #设置工作目录

#读取基因表达文件,并对数据进行处理
rt=read.table(expFile, header=T, sep="\t", check.names=F)
rt=as.matrix(rt)
rownames(rt)=rt[,1]
exp=rt[,2:ncol(rt)]
dimnames=list(rownames(exp),colnames(exp))
data=matrix(as.numeric(as.matrix(exp)),nrow=nrow(exp),dimnames=dimnames)
data=avereps(data)

#读取基因文件,获取免疫检查点相关基因的表达量
gene=read.table(geneFile, header=F, sep="\t", check.names=F)
sameGene=intersect(row.names(data), as.vector(gene[,1]))
data=t(data[sameGene,])
data=log2(data+1)
```

```
#删除正常样品
group=sapply(strsplit(row.names(data),"\\-"),"[",4)
group=sapply(strsplit(group,""),"[",1)
group=gsub("2","1",group)
data=data[group==0,]
row.names(data)=gsub("(.*?)\\-(.*?)\\-(.*?)\\-(.*?)\\-.*",                    "\\1\\-\\2\\-\\3",
row.names(data))
data=avereps(data)

#合并数据
risk=read.table(riskFile, sep="\t", header=T, check.names=F, row.names=1)
sameSample=intersect(row.names(data),row.names(risk))
rt1=cbind(data[sameSample,],risk[sameSample,])
rt1=rt1[,c(sameGene,"risk")]

#提取显著差异的基因
sigGene=c()
for(i in colnames(rt1)[1:(ncol(rt1)-1)]){
 if(sd(rt1[,i])<0.001){next}
 wilcoxTest=wilcox.test(rt1[,i] ~ rt1[,"risk"])
 pvalue=wilcoxTest$p.value
 if(wilcoxTest$p.value<0.05){
        sigGene=c(sigGene, i)
 }
}
sigGene=c(sigGene, "risk")
rt1=rt1[,sigGene]

#把数据转换成 ggplot2 输入文件
rt1=melt(rt1,id.vars=c("risk"))
colnames(rt1)=c("risk","Gene","Expression")

#设置比较组
group=levels(factor(rt1$risk))
rt1$risk=factor(rt1$risk, levels=c("low","high"))
comp=combn(group,2)
my_comparisons=list()
for(j in 1:ncol(comp)){my_comparisons[[j]]<-comp[,j]}

#绘制箱线图
boxplot=ggboxplot(rt1, x="Gene", y="Expression", fill="risk",
                          xlab="",
                          ylab="Gene expression",
```

```
                                  legend.title="Risk",
                                  width=0.8,
                                  palette = c("#0066FF", "#FF0000") )+
                                  rotate_x_text(50)+
        stat_compare_means(aes(group=risk),
        method="wilcox.test",
        symnum.args=list(cutpoints=c(0, 0.001, 0.01, 0.05, 1), symbols=c("***", "**", "*", "ns")), label="p.signif")

#输出图片
pdf(file="checkpoint.diff.pdf", width=8, height=5)
print(boxplot)
dev.off()
```
